# Characterization of distinct strains of an aphid-transmitted ilarvirus (Fam. *Bromoviridae*) infecting different hosts from South America

**DOI:** 10.1016/j.virusres.2020.197944

**Published:** 2020-06

**Authors:** Rocio Silvestre, Segundo Fuentes, Roger Risco, Alfredo Berrocal, Ian Adams, Adrian Fox, Wilmer J. Cuellar, Jan Kreuze

**Affiliations:** a Virology Laboratory, Crop and Systems Science Division, International Potato Center (CIP), Lima, Peru; b Department of Plant Sciences, University of Idaho, Moscow, ID, USA; c Universidad Nacional Agraria la Molina, Lima, Peru; d Facultad de Agronomía, Universidad de Buenos Aires, Argentina; e Fera Science Ltd, Sand Hutton, York, YO41 1LZ, United Kingdom; f Virology Laboratory, International Center for Tropical Agriculture (CIAT), Cali, Colombia

**Keywords:** *Ilarvirus*, *Alfamovirus*, *Solanum*, *Smallanthus*, Potato yellowing virus, HTS, NGS

## Abstract

Potato yellowing virus (PYV, original code SB-22), an unassigned member of the Genus *Ilarvirus* Family *Bromoviridae*, has been reported infecting potatoes in Peru, Ecuador and Chile. It is associated with symptomless infections, however yellowing of young leaves has been observed in some potato cultivars. Thirteen potato and yacon isolates were selected after routine screening of CIP-germplasm and twenty-four were identified from 994 potato plants collected in Peru whereas one was intercepted from yacon in the UK. These isolates were identified using high throughput sequencing, ELISA, host range and RT-PCR. Here we report the sequence characterization of the complete genomes of nine PYV isolates found infecting *Solanum tuberosum*, four complete genome isolates infecting *Smallanthus sonchifolius* (yacon), and in addition 15 complete RNA3 sequences from potato and partial sequences of RNA1, 2 and 3 of isolates infecting potato and yacon from Ecuador, Peru and Bolivia. Results of phylogenetic and recombination analysis showed RNA3 to be the most variable among the virus isolates and suggest potato infecting isolates have resulted through acquisition of a movement protein variant through recombination with an unknown but related ilarvirus, whereas one yacon isolate from Bolivia also had resulted from a recombination event with another related viruses in the same region. Yacon isolates could be distinguished from potato isolates by their inability to infect *Physalis floridana*, and potato isolates from Ecuador and Peru could be distinguished by their symptomatology in this host as well as phylogenetically. The non-recombinant yacon isolates were closely related to a recently described isolate from *Solanum muricatum* (pepino dulce), and all isolates were related to *Fragaria chiloensis latent virus* (FCiLV) reported in strawberry from Chile, and probably should be considered the same species. Although PYV is not serologically related to *Alfalfa mosaic virus* (AMV), they are both transmitted by aphids and share several other characteristics that support the previous suggestion to reclassify AMV as a member in the genus *Ilarvirus*.

## Highlights

• Thirteen complete genomes and 25 partial sequences of PYV from potato and yacon collected in Ecuador, Peru, Bolivia and a UK interception.
• Analysis suggests potato isolates originated via acquisition of the movement protein from a related virus through recombination.
• Most yacon isolates and potato isolates from Peru and Ecuador could be distinguished through infectivity and symptoms in different hosts.

## 1 Introduction

Potato yellowing virus (PYV; genus: *Ilarvirus*, family: *Bromoviridae*) was originally isolated (coded as SB-22) from Peruvian potato landrace “Ticahuasi” (*Solanum tuberosum* Linneo subsp*. tuberosum x* subsp*. andigenum* Juz. & Bukasov) grown in the Ica valley, south of Lima in Peru. Although related to *Alfalfa mosaic virus* (AMV), a member of the genus *Alfamovirus* in the family *Bromoviridae*, due to a similar particle morphology, host range, and aphid-transmission the symptoms induced by SB-22 were generally milder and it has no known local lesion hosts. PYV is transmitted by sap and by *Myzus persicae* (Sulz.) in a non-persistent manner. It induces mild yellowing in the leaves or remains symptomless in potato. Furthermore, unlike AMV, PYV has been shown to be transmitted through pollen and botanical seed. Therefore, PYV and AMV were considered as two distinct viruses. Although PYV has been mentioned in scientific publications (Duarte et al., 2016; Fuentes and Jayasinghe, 1993; Knierim et al., 2019; Silvestre et al., 2011; Sivaprasad et al., 2015; Valkonen et al., 1992b), it has not yet been formally accepted as a virus species by the International Committee for Taxonomy of Viruses.

PYV has bacilliform particles and it can be mechanically inoculated to the indicator plant *Physalis floridiana* Linneo in which it induces severe mosaic and deformation of leaves. PYV has been found infecting potatoes in Peru, Chile and Ecuador (Fuentes, 1992; Silvestre et al., 2011; Valkonen et al., 1992a, b) and infecting pepper (*Capsicum annum* Linneo) in Ecuador (Sivaprasad et al., 2015). Morphology of PYV particles and its host range are most similar with those of AMV, another virus frequently infecting potatoes in Peru and Chile (Valkonen et al., 1992b) and with *Fragaria chiloensis latent virus* (FCiLV), a virus reported infecting wild and cultivated strawberry and found along the west coast of the Americas (Martin and Tzanetakis, 2006; Spiegel et al., 1993; Tzanetakis and Martin, 2005). New sequence analysis suggests they are members of genus *Ilarvirus* (Boulila, 2009; Codoner and Elena, 2006, 2008). Indeed, the principal reason for maintaining AMV in a separate genus from Ilarviruses is that it is transmitted in a non-persistent manner by aphids, and ilarviruses are reportedly transmitted through pollen and thrips (Card et al., 2007; King et al., 2012). Partial sequence information of the conserved Helicase (Hel) domain of PYV isolates groups these sequences with those of the genus *Ilarvirus* closely related to FCiLV with 77 % nucleotide and 85 % amino acid sequence identity and indicated that geographically distinct isolates of PYV exist (Silvestre et al., 2011). Recently a complete nucleotide sequence of a PYV isolate infecting pepino in Germany was reported and shared between 78 %–87 % amino acid identity of open reading frames (ORF) when is compared with FCiLV (Knierim et al., 2019)

The *Bromoviridae* have a tripartite positive-strand RNA genome and infect a wide range of hosts including herbaceous plants, shrubs and trees which makes them one of the most important families of plant viruses. The family is divided into six genera: *Alfamovirus*, *Anulavirus*, *Bromovirus*, *Cucumovirus*, *Oleavirus* and *Ilarvirus*. The genus *Ilarvirus* has the most member species in the family and is further divided into four sub-groups (King et al., 2012). Ilarviruses cause diseases of economic importance in *Citrus, Humulus, Malus, Prunus, Rosa* and *Rubus* spp., affecting plant growth, and fruit yield and maturity (Tzanetakis et al., 2008; Uyemoto and SFmon W, 1992). On the other hand, the alfamovirus AMV has an extremely broad host range affecting many different crops. The genome of ilarvirus (and *Bromoviridae* in general) encodes four to five proteins encoded in 3 RNA molecules which contain 5′ cap structures and no poly-A tail at their 3′ end: RNA1 is monocistronic and encodes for methyltransferase (Mtr) and Hel signature motifs. RNA2 can be mono or bicistronic encoding an RNA-dependent-RNA polymerase (RdRp) which is also known as 2a protein. In some species of subgroup 1 and 2 of the genus *Ilarvirus*, a second gene is present in RNA2 (Xin et al., 1998). This gene, named 2b, is expressed *in vivo* through a subgenomic mRNA and, similar to the 2b protein of cucumoviruses, is thought to be involved in suppression of RNA silencing (Brigneti et al., 1998) and in the cell to cell movement (Xin et al., 1998). RNA3 encodes the movement protein (MP) and coat protein (CP). While the MP is expressed directly from this RNA, CP synthesis occurs via a monocistronic and subgenomic RNA. In addition to its structural role, the CP of ilarviruses is required for virus movement and genome activation (Jaspars, 1999; Neeleman et al., 2004). The requirement of the CP for genome activation is shared by all ilarviruses and AMV but is not found in other members of the family (Bol, 1999). The total genome size of ilarviruses is ∼8 kb on average and genomic RNAs are packaged in separate virions.

Yacon [*Smallanthus sonchifolius* (Poepp. and Endl.) H. Robinson; Asteraceae] is a perennial herb originally cultivated in the Andean highlands, from southern Colombia to northern Argentina for its edible roots as well as medicinal purposes. Yacon roots have a high amount of fructo-oligosaccharides (40–70% dry weight), that are not metabolized in the digestive tract of humans (Ovesna et al., 2018). Currently, yacon is grown in Brazil, Korea, Japan, Mexico, United States, Czech Republic, Russia, Estonia, Taiwan, New Zealand and China (Fernández et al., 2006; Ovesna et al., 2018). In South America yacon is very common in Bolivia in the departments of Tarija, Chuquisaca, Cochabamba and La Paz. In Peru, the largest germplasm diversity is found in Cuzco, Puno, Cajamarca and the area close to the Ecuadorian border. In Ecuador, yacon is predominantly grown in the southern provinces of Loja, Azuay and Cañar (Hernann and Heller, 1997).In this study we identify PYV from potato and yacon samples from CIPs germplasm collection, almost a thousand potato samples collected from production fields in Peru and yacon intercepted in the UK by high throughput sequencing, ELISA, PCR and, biological characterization of some of them. Results are discussed in relation to relative economic importance, sequence variation, evolutionary aspects and taxonomical implications.

## 2 Materials and methods

### 2.1 Plant material, growing conditions, host range infection

The type isolate of PYV [SB-22], was obtained from the International Potato Center (CIP) virus collection, where it has been maintained in *Physalis floridiana* Linneo since its original isolation from potato in 1985 (Fuentes, 1992; Fuentes and Jayasinghe, 1993) and was characterized by small RNA sequencing and confirmed by PCR sequencing as described below. Besides SB-22, the current study included four groups of samples from which PYV was detected (Table 1):

**Table 1:** List of PYV isolates from potato and yacon analyzed in this work. (*) partial genomes/ORFs (**) potato isolates with long 5′ *UTR sequence in RNA3.*

Sample Group	Isolate	Host	Cultivar	CIP Accession number	Genebank Accession Number	Country of Origin	Reference	# in Fig 2
Reference isolate	SB-22	*Solanum tuberosum*	Ticahuasi	PYV	MG672020 (RNA1)*	Peru	Silvestre et al., 2011	–
					MG672021 (RNA2)*			
					MG672022 (RNA3)			
Group 1	Jin-5P	*Solanum tuberosum*	Breeding line	CIP395017.229	MG672023 (Mtr-Hel)*	Peru	This work	1
					MG672024 (RdRp)*			
					MG672025 (MP-CP)*			
	Jin-7P	*Solanum tuberosum*	Breeding line	CIP396008.104	MG672026 (Mtr-Hel)*	Peru	This work	2
					MG672027 (RdRp)*			
					MG672028(MP_CP)*			
	Jin-11P	*Solanum tuberosum*	Breeding line	CIP396029.205	MG676881 (Mtr-Hel)*	Peru	This work	3
					MG676882 (RdRp)*			
					MG676883 (MP-CP)*			
	Loj-22E	*Solanum phureja*	Chaucha amarilla	CIP706784	MH760373 (Mtr-Hel)*	Ecuador	Silvestre et al., 2011	4
					MH760374 (RdRp)*			
					MH760375 (MP-CP)*			
	Loj-23E	*Solanum phureja*	Chaucha negra ojona	CIP706787	MH760370 (Mtr-Hel)*	Ecuador	Silvestre et al., 2011	5
					MH760371 (RdRp)*			
					MH760372 (MP-CP)*			
	Azu-24E	*Solanum phureja*	Cuica	CIP706822	MH767083 (Mtr-Hel)*	Ecuador	Silvestre et al., 2011	6
					MH767084 (RdRp)*			
					MH767085 (MP-CP)*			
	Can-25E	*Solanum phureja*	Chaucha tomate	CIP706828	MH767080 (Mtr-Hel)*	Ecuador	Silvestre et al., 2011	7
					MH767081 (RdRp)*			
					MH767082 (MP-CP)*			
	Yacon Coc-919	*Smallanthus sonchifolius*	Yacon	CIP205030	MH760368 (RdRp)*	Bolivia	This work	33
					MH760367 (CP)*			
	Yacon Lima-5027	*Smallanthus sonchifolius*	Yacon	CIP205004	MH760369 (RdRp)*	Peru	This work	32
					MH760366 (CP)*			
Group 2	Apu-10	*Solanum tuberosum*	–	–	MN527469 (RNA1)	Peru	This work	8
					MN527470 (RNA2)			
					MN527471 (RNA3)			
	Apu-10A	*Solanum tuberosum*	–	–	MN527472 (RNA1)	Peru	This work	9
					MN527473 (RNA2)			
					MN527474 (RNA3)			
Group 2	Cca-059	*Solanum tuberosum*	Yungay	–	MN527517(RNA3)**	Peru	This work	10
	Czo-096	*Solanum tuberosum*	Canchan	–	MN527509 (RNA3)	Peru	This work	11
	Czo-097	*Solanum tuberosum*	Canchan	–	MN527508 (RNA3)	Peru	This work	12
	Czo-099	*Solanum tuberosum*	Canchan	–	MN527506 (RNA3)	Peru	This work	13
	Czo-118	*Solanum tuberosum*	Cica	–	MN527475 (RNA1)	Peru	This work	14
					MN527476 (RNA2)			
					MN527477 (RNA3)			
	Czo-124	*Solanum tuberosum*	Yungay	–	MN527512 (RNA3)	Peru	This work	15
	Hco-024	*Solanum tuberosum*	Canchan Blanca	–	MN527519(RNA3)**	Peru	This work	16
	Hco-030B	*Solanum tuberosum*	Canchan Blanca	–	MN527493 (RNA1)	Peru	This work	17
					MN527494 (RNA2)			
					MN527495(RNA3)**			
	Hua-025	*Solanum tuberosum*	Ccompis Huayro	–	MN527514 (RNA3)	Peru	This work	18
	Hua-029	*Solanum tuberosum*	Ccompis Huayro	–	MN527478 (RNA1)	Peru	This work	19
					MN527479 (RNA2)			
					MN527480 (RNA3)			
	Hua-060A	*Solanum tuberosum*	–	–	MN527481 (RNA1)	Peru	This work	20
					MN527482 (RNA2)			
					MN527483 (RNA3)			
	Ica-086	*Solanum tuberosum*	Canchan	–	MN527505 (RNA3)	Peru	This work	21
	Ica-087	*Solanum tuberosum*	Canchan	–	MN527511 (RNA3)	Peru	This work	22
	Jin-100B	*Solanum tuberosum*	Andina	–	MN527513 (RNA3)	Peru	This work	23
	Jin-116	*Solanum tuberosum*	Yungay	–	MN527518(RNA3)**	Peru	This work	24
	Jin-Hua-146	*Solanum tuberosum*	Andina	–	MN527484 (RNA1)	Peru	This work	25
					MN527485 (RNA2)			
					MN527486 (RNA3)			
Group 2	Jin-Hua-148	*Solanum tuberosum*	Andina	–	MN527515 (RNA3)	Peru	This work	26
	Jin-Hua-149	*Solanum tuberosum*	Andina	–	MN527507 (RNA3)	Peru	This work	27
	Jin-Hua-152	*Solanum tuberosum*	Yungay	–	MN527487 (RNA1)	Peru	This work	28
					MN527488 (RNA2)			
					MN527489 (RNA3)			
	Jin-165	*Solanum tuberosum*	Yungay	–	MN527516 (RNA3)**	Peru	This work	29
	Lim-099	*Solanum tuberosum*	Unica	–	MN527510 (RNA3)	Peru	This work	30
	Pun-015	*Solanum tuberosum*	Ccompis	–	MN527490 (RNA1)	Peru	This work	31
					MN527491 (RNA2)			
					MN527492 (RNA3)			
Group 3	Yacon Anc-205011	*Smallanthus sonchifolius*	Yacon	CIP205011	MN527502 (RNA1)	Peru	This work	34
					MN527503 (RNA2)			
					MN527504 (RNA3)			
	Yacon Caj-205023	*Smallanthus sonchifolius*	Yacon	CIP205023	MN527496 (RNA1)	Peru	This work	35
					MN527497 (RNA2)			
					MN527498 (RNA3)			
	Yacon Coc-205025	*Smallanthus sonchifolius*	Yacon	CIP205025	MN527499 (RNA1)	Bolivia	This work	36
					MN527500 (RNA2)			
					MN527501 (RNA3)			
Group 4	PYV York	*Smallanthus sonchifolius*	Yacon	–	MN548138 (RNA1)	UK	This work	–
					MN548139 (RNA3) MN548140 (RNA2)			
_	FCiLV	*Fragaria chiloensis*	Chilean Strawberry	–	AY682102 (RNA1)	Chile	Tzanetakis and Martin, 2005	–
					AY707771 (RNA2)			
					AY707772 (RNA3)			
_	DSMZ PV-0706	*Solanum muricatum*	–	–	MH937418 (RNA1)	unknown	Knierim et al., 2019	–
					MH937419 (RNA2)			
					MH937420 (RNA3)			

The first set of samples (Group 1) consisted of PYV-infected potato accessions that were identified by double antibody sandwich - enzyme Linked Immunosorbent Assay (DAS-ELISA) during routine virus indexing of germplasm between 2006–2011, and also two *in vitro* yacon plantlets from Peru and Bolivia that were identified by ELISA on nitrocellulose membranes (NCM-ELISA) from CIPs germplasm collection in 2011. This included virus isolates from three breeding lines and native potatoes *Solanum phureja* Juz. & Bukasov cultivars ‘Chaucha tomate’, ‘Chaucha negra ojona’, ‘Chaucha amarilla’ and ‘Cuica’, that were collected from the provinces of Cañar, Loja and Azuay in the south of Ecuador (Silvestre et al., 2011) and native yacon that were collected from the departments of Cochabamba-Bolivia and Lima-Peru (Table 1). These isolates, were molecularly characterized by sequencing of PCR fragments and biologically characterized by host range infection with the following hosts: *Gomphrena globosa*, Linneo (Family *Amaranthaceae*); *Chenopodium murale* Linneo and *C. quinoa* Willd (Family *Chenopodiaceae*); *Nicotiana benthamiana* Domin, *N. bigelovii* Pursh x *N clevelandii* Gray, *N. debneyii* Domin, *N. glutinosa* Linneo, *N. tabacum* Linneo cv. White Burley, *Lycopersicon esculentum* Mill Rutgers, *Physalis floridana* Linneo and *Datura stramonium* Linneo*, Solanum tuberosum* Linneo cultivars “Yungay” and “Amarillis” (Family *Solanaceae*). In the case of two PYV isolates from yacon, an additional host *N. occidentalis* Wheeler was used. Plants (three replicates) were mechanically inoculated using extracts of a bulk of leaves, obtained from different positions of the infected plants, in phosphate buffer with carborundum. Leaf extracts were gently applied to basal leaves using cotton swabs. All plants were maintained in an insect–proof screenhouse at 19 °C. Symptoms under these conditions were recorded around 4 weeks post inoculation and samples tested by DAS-ELISA, NCM-ELISA or RT-PCR in 3–4 replicates.

A second set of samples consisted of leaves from 994 individual potato plants collected between 2016–2018 in the northern, central and southern Andean highlands of Peru. Symptoms were recorded for each plant by photographs. Each sample was placed in a separate labelled paper filter bag, 9 of which were put together in a zip-lock plastic bag filled with 100 g of dehydrated silica-gel for rapid desiccation. Silica-gel was changed after 24−48 hours and samples were brought to CIP facilities for small RNA sequencing and assembly (sRSA) as described below.

A third set of samples consisted of 14 in-vitro yacon plants from the germplasm collection of CIP. Entire in-vitro plantlets were used to extract RNA for sRSA (see section 2.3). These plants were also grown out in a greenhouse and biologically characterized by host range as described for group 1 plants.

A fourth set of samples consisted of yacon plants collected in the United Kingdom. These yacon tubers were sourced as part of a project to assess the plant health risk to the European Union from the internet trade in Andean root and tuber crops, following an interception of viruses from *Ullucus tuberosus* (Fox et al., 2019). The yacon tubers were sourced from a vendor in Poland via an internet shopping site and brought to the Fera laboratory where they were tested using ribosomal RNA depleted total RNA and assembly as described below (section 2.3).

*Fragaria chiloensis* (L.) Mill (Family *Rosaceae*) infected with ilarvirus was kindly provided by Dr Robert R. Martin from Horticultural Crops Research Laboratory, USDA-ARS.

### 2.2 Virus detection by ELISA

Group 1 (Table 1) *in vitro* potato and yacon accessions were screened by DAS- and NCM-ELISA respectively, and results confirmed by sequencing of PCR-amplified viral fragments. Group 2, desiccated leaf samples and group 3 greenhouse grown samples in which PYV was identified by sRSA, were tested by NCM- and DAS-ELISA respectively to confirm infections. The yacon tubers from group 4 were planted in the glasshouses at Fera and grown out. Following the identification of PYV by ribo-depleted RNA sequencing the presence of PYV was confirmed with antiserum kindly supplied by Dr. Wulf Menzel, DSMZ, Germany, raised against the German isolate of PYV (Knierim et al., 2019).

Except for group 4 samples, an antiserum raised against the Peruvian PYV isolate SB-22 (Fuentes and Jayasinghe, 1993) was used for ELISA tests. Antiserum against FCiLV was kindly provided by Dr. Robert R. Martin. For DAS-ELISA, fresh leaf-material was collected and ground in 1/10 w/v PBS-T buffer (3.2 mM Na_2_HPO_4_, 0.5 mM KH_2_PO_4_, 1.3 mM KCl, 135 mM NaCl, 0.05 % Tween 20, pH 7.4), containing 2 % PVP-40 and 1 % ovoalbumin. 100 u L of leaf extract were then added to ELISA plate wells that were previously coated (4 h at 37 °C) with a polyclonal anti-PYV antiserum (IgG) diluted 1/1500 with coating buffer (15 mM Na_2_CO_3_, 35 mM NaHCO_3_, 3 mM NaN_3_, pH 9,6). The plates were incubated at 4 °C overnight. After three washings with PBS-T, 100 u L of 1/500 dilution (in PBS containing 0.2 % PVP40, 0.1 % ovoalbumin) of anti-PYV IgG alkaline phosphatase conjugate IgG were added and the plates incubated again at 37 °C for 3 h. After three washings with PBS-T, substrate (0.5 mg/mL p-nitrophenyl phosphate tablets (SIGMA) in diethanolamine) was added and absorbance lectures were taken after 30 and 60 min of reaction at room temperature. For NCM-ELISA the membranes were pre-soaked in TBS buffer (Tris base 20 mM, NaCl 500 mM, pH 7.5). 200 mg of fresh leaf material (for group 1) or 40 mg of desiccated leaf material (for group 2) were ground in extraction buffer (TBS containing 0.2 % sodium sulfite) and 20 u L were applied to the membrane. After blocking for 1 h at room temperature in blocking solution (TBS containing 2 % powder milk and 2 % Triton X-100), the membranes were washed one time in TBS-T (TBS plus 0.05 % Tween-20) and incubated overnight at room temperature with a 1/500 dilution of the PYV antiserum in TBS with 2 % powder milk. Next day after three washings in TBS-T, the secondary antibody (goat anti rabbit-alkaline phosphatase) was added diluted 1/500 in buffer TBS with 2 % powder milk, incubated for 1 h at room temperature and washed three times in TBS-T. Color reactions were visible after adding developing solution containing NBT and BCIP in substrate buffer (100 mM Tris base, 100 mM NaCl, 5 u M MgCl_2_.6H_2_O) and incubation for 30 min.

### 2.3 RNA extraction, library preparation and reverse transcription PCR

Total RNA was extracted from 250 mg of fresh leaf tissue using TRIZOL (Invitrogen) following supplier instructions. For the third group (yacon accessions) adjustments were made in the amount of tissue, increasing from 250 mg to 400 mg, to obtain sufficient amount of RNA, the proportion of tissue: trizol was maintained. RNA quality and quantity were checked by using agarose gel electrophoresis and spectro-photometry (Nanodrop ND-1000, Thermo Fisher Scientific, Waltham, MA, USA).

For initial sequencing of the PYV [SB-22] genome total RNA was extracted from an infected indicator host *P. floridiana* Linneo using Trizol (Invitrogen) and small RNA (sRNA) isolated according the procedure previously reported (Kreuze et al., 2009). The sample was bulked together with sRNA from 17 other samples of different crops, to save cost, and then sent to a provider for library preparation and sequencing on llumina Genome Analyzer (Fasteris Life Sciences SA, Plan-les-Ouates, Switzerland).

In the case of field samples of potato collected during 2016–2018 and yacon accessions from the germplasm bank (groups 2 and 3, see Table 1), small RNA libraries were prepared from individual samples according to the protocol previously reported (Chen et al., 2012), bulking together 48 indexed samples per lane of sequencing on a Illumina HiSeq4000 by Fasteris.

For cDNA synthesis 1 ug of total RNA and random hexamer primers were used in a total reaction mix of 20 u L using 200 U of M-MLV reverse transcriptase (Invitrogen). After incubation at 37 °C for 50 min, the reaction was diluted 1:10 with sterile water and 5 u L were used for PCR using 2X Phusion polymerase ready-made master mix (Finzymes, Finland). For initial PCR confirmation of infection in individual plants of group 1 (Table 1), degenerate primers Ilar1F5: 5′-GCN GGW TGY GGD AAR WCN AC-3′ and Ilar1R7: 5′-AMD GGW AYY TGY TYN GTR TCA CC-3′ were used as described previously (Untiveros et al., 2010) to amplify a conserved region of the Hel domain of PYV RNA1. To verify sequence of the complete genome of PYV [SB-22], specific primers (Table 2) were designed using as reference the complete genome of FCiLV and the PYV [SB-22] consensus sequence assembled from reads obtained by Illumina sequencing of small RNA. To amplify the 3´ extreme of the PYV [SB-22] genome, Modban linker and Ban One Primers were used and for the 5´extreme the Ban Two Linker and Ban Two Primers were used (Table 2). The primers designed were also used to amplify and complete the partial genomes of potato and yacon isolates from group 1.

**Table 2:** Primers designed from the consensus sequence of PYV [SB-22] and FCiLV to complete the genome of PYV isolates and primers used to primers used to amplify the 3´UTR and 5´UTR extremes of PYV [SB-22] genome.

Primer Name	Primer sequence (5′-3′)	Type of RNA	ORF
PYV1F1	TTGAATATTTCGTTTCAACTCTCGG	RNA1	Mtr-Hel
PYV1F2	GCTGATCCCGAGTTGTTTGTACC	RNA1	Mtr-Hel
PYV1R1	TCATATTCACCGCGATACGTAGG	RNA1	Mtr-Hel
PYV1F3	ATTGTGGTCGTTCCTGTGCTTGG	RNA1	Mtr-Hel
PYV1R2	CATCGGCCCTCTCTAGCTCAA	RNA1	Mtr-Hel
PYV1F4	CTGCTGGGTCCCATCCTACTATGG	RNA1	Mtr-Hel
PYV1R3	CTACCATCAAGAAGCGGACAGCAG	RNA1	Mtr-Hel
PYV2F1	CATGCGGTTCGTCGAGCATG	RNA2	RdRp
PYV2F2	AGGACATGTTTGTTGATCCGATATG	RNA2	RdRp
PYV2R1	CATGCTCGACGAACCGCATG	RNA2	RdRp
PYV2R2	GTTATAGTGGCGGGTAAGGGTCTCTC	RNA2	RdRp
PYV2F4	GCCTGCTATGTGTGCCTTCAATAATG	RNA2	RdRp
PYV3F1	CAAGTCTTTGTGAGTGTCAGATTGTG	RNA3	MP
PYV3F2	ATGGCTTTTTGTAATGTATGCG	RNA3	CP
PYV3F3	ATACCGCGTTTGTGGCGAATACAG	RNA3	CP
PYV3R1	GTGCGTCGGGTCCGTTTATCTC	RNA3	MP and CP
PYV3R2	GACTGCAAAGCCAAAGACTCAATCG	RNA3	MP
PYV-123R	GCCATCCTTTCGGGCATTAATTC	RNA1, RNA2, RNA3	Mtr-Hel/RdRp/CP
Ban One	ATTGATGGTGCCTACAG	3 UTR	
Modban linker	rAppCTGTAGGCACCATCAAT/3ddC/	3 UTR	
Ban Two Primer	ATCGTAGGCACCTGAAA	5 UTR	
Ban Two linker	ATCGTrArGrGrCrArCrCrUrGrArArA	5 UTR	

The group 4 sample was processed as described in (Adams et al., 2014). RNA extracted using the RNAeasy kit (Qiagen, UK) and plant leaf ribo-depleted total RNA libraries produced using the Scriptseq Complete kit (Illumina, UK). The library was indexed and pooled with other Scriptseq libraries prior to sequencing on an Illumina MiSeq using a V3 kit (Illumina, UK).

### 2.4 Sequence analysis

Reads (18 364 310) obtained by Illumina sequencing of small interfering RNAs (siRNAs) (available from https://research.cip.cgiar.org/confluence/download/attachments/85165099/GAF13_21−24.fastq.gz?api = v2), obtained from a bulk sample including PYV [SB-22], were assembled using the program VELVET (Zerbino and Birney, 2008) and MAQ (Li et al., 2008), and the contigs obtained were compared with the Genbank database using BLASTN and BLASTX. As an additional strategy the genome sequence of FCiLV was used as template to align sRNA reads using MAQ, VELVET and NovoAlign software (http://www.novocraft.com/products/novoalign/). Contigs matching ilarvirus sequences were mapped to the closest common relative ilarvirus genome and primers were designed accordingly. Partial sequence information (contigs) obtained from sequencing of PYV [SB-22] infected indicator plant was used for design of specific primers spanning the complete genome of the virus (Table 2). PCR products were cloned into plasmid vector pGEM-T easy (Promega), following standard procedures and sent for Sanger sequencing to Macrogen (Korea). Contigs and sequences obtained by RT-PCR were assembled using the program ContigExpress contained in the VectorNTI Advance v9.1 package (Invitrogen) and Seqman v16.0 contained in the DNASTAR package. Alignment of sequences were done using CLUSTAL v.2.0 (Larkin et al., 2007) and phylogenetic analyses by MEGA v7.0 (Kumar et al., 2016). For samples from group 2 (Fuentes et al., 2019) (data available from http://potpathodiv.org/index.html), and 3 (data available from https://data.cipotato.org/dataset.xhtml?persistentId = doi:10.21223/ANSPIN), small RNA sequences were cleaned and analyzed using VirusDetect v1.6 (Zheng et al., 2017) to identify all viruses infecting the plants, and samples in which PYV was identified were selected for further analysis. Using the Geneious R11.1.3 software package, the PYV contigs produced by VirusDetect were extracted for each positive sample and a consensus generated. Then the small RNAs were mapped back to the consensus to confirm the quality of the assemblies and make any corrections as necessary.

851,454 300bp paired reads (NCBI short read archive biosample accession SAMN12993632) were obtained from the group 4 sample were quality trimmed, assembled and the resulting contigs analyzed by BLAST as described in Adams et al. (2014).

Recombination analysis was carried out using RDP, GENECONV, BootScan, MaxChi, Chimaera, SIScan and 3Seq methods implemented in RDP4 (Martin et al., 2015). Alignments of the complete RNA3 sequence of potato, yacon and pepino isolates of PYV and FCiLV were used for recombination analysis (supplementary Table 1). The analysis was done using default settings and a Bonferroni correction *P*-value of 0.05. The recombination breakpoints detected by a minimum of seven methods within the program were considered significant.

## 3 Results

### 3.1 Host range, symptoms and serological detection

**Fig. 1:**
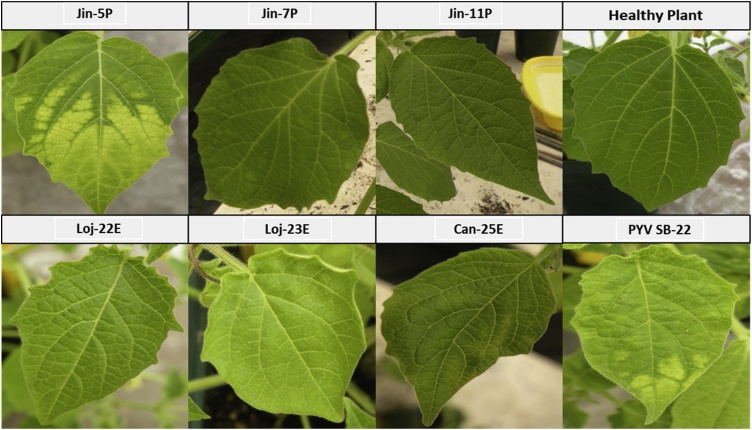
Symptoms induced by the PYV isolates mechanically transmitted in *Physalis floridiana* Linneo.

Thirteen PYV isolates (SB-22 and group 1, and group 3) were biologically characterized in this study, including 5 from Yacon. As previously reported PYV [SB-22] induced leaf mosaic and chlorotic spots symptoms in *P. floridiana* Linneo. However, this isolate was associated mostly with symptomless infection in potato (*Solanum* sp.) (Fuentes, 1992; Fuentes and Jayasinghe, 1993). None of the isolates analyzed in group 1 showed visible symptoms in their respective potato or yacon hosts from CIP germplasm. Host range analyses resulted in symptomless infections in most of the indicator plants used for potato isolates but not the yacon isolates. Symptoms showed a difference in *P. floridiana*: potato isolates from Peru (all isolated from *Solanum tuberosum* Linneo) were mostly associated to clear mosaic symptoms while isolates from Ecuador (isolated from *Solanum phureja* Juz & Bukasov) were mostly associated to mild leaf mosaic and mild upward leaf rolling (Fig. 1). These indicator plants were tested by DAS-ELISA and positive reaction was detected in *P. floridana* Linneo inoculated with all potato isolates. In contrast, the two group 1 PYV isolates from yacon (from Bolivia and Peru) were unable to infect *P. floridiana*, although this was based on only three plants and can therefore not be considered conclusive (Table 3). When tested by NCM-ELISA systemic infections of PYV isolates from yacon were detected in *D. stramonium* for both isolates, and *C. quinoa* and by RT-PCR in potato cv. Yungay for isolate Coc-919 (Bolivian isolate). Yacon samples from group 3 in which PYV was detected showed apical leaf deformation and mild mosaic (Anc-205011) or interveinal chlorosis in the leaves (Coc-205025 & Caj-205023). Yacon plants of Anc-205011 and Caj-205023 showed mild reaction to PYV antisera while Coc-205025 was negative. Mechanical inoculation to indicator host range resulted in symptoms of stunting and severe mottle, and positive ELISA reaction only for isolate Anc-205011 in *P. floridana* (not shown).

**Table 3:** Symptoms expressed in, and ELISA and RT-PCR results from, plants mechanically inoculated with PYV isolates from potato and yacon to an indicator host range.

	Isolates									
**Original plants**	**Jin-5P**	**Jin-7P**	**Jin-11P**	**Loj-22E**	**Loj-23E**	**Can-25E**	**Yacon Coc-919**	**Yacon Lim-5027**	**Control (SB-22)**	**Mock**
**DAS-ELISA**	+	+	+	+	+	+	N	N	+	–
**NCM-ELISA**	N	N	N	N	N	N	+	+	+	–
**RT-PCR**	+	+	+	+	+	+	+	+	+	–
**Host Range**										
*Gomphrena globosa*	N	S^+^	N	S^+^	S^+^	S^+^	S^¯^	S^¯^	S^+^	–
*Chenopodium murale*	S^+^	S^+^	S^+^	S^+^	S^+^	S^+^	S^¯^	S^¯^	S^+^	–
*Chenopodium quinoa*	S^+^	S^+^	S^+^	S^+^	S^+^	S^+^	S^+^	S^¯^	S^+^	–
*Nicotiana benthamiana*	S^+^	S^+^	S^+^	S^+^	S^+^	S^+^	S^¯^	S^¯^	S^+^	–
*N. bigelovii x N clevelandii*	S^+^	S^+^	S^+^	S^+^	S^+^	S^+^	S^¯^	S^¯^	S^+^	–
*N. debneyii*	S^+^	S^+^	S^+^	S^+^	S^+^	S^+^	S^¯^	S^¯^	S^+^	–
*N. glutinosa*	S^+^	S^+^	S^+^	S^+^	S^+^	S^+^	S^¯^	S^¯^	S^+^	–
*N. tabacum cv. White Burley*	S^+^	S^+^	S^+^	S^+^	S^+^	S^+^	S^¯^	S^¯^	S^+^	–
*N. occidentalis*	N	N	N	N	N	N	S^¯^	S^¯^	S^+^	–
*Datura stramonium*	S^+^	S^+^	S^+^	S^+^	S^+^	S^+^	S^+^	S^+^	S^+^	–
*Lycopersicon esculentum Rutgers*	S^+^	S^+^	S^+^	S^+^	S^+^	S^+^	S¯	S¯	S^+^	–
*Physalis floridana*	SM, CS^+/+^	CS^+/+^	MM^+/+^	MM, LR^+/+^	LR^+/+^	MM^+/+^	S¯	S¯	SM, CS^+/+^	–
*Solanum tuberosum* Yungay	S^¯^	S^¯^	S^¯^	S^¯^	S^¯^	S^¯^	S^−/+^	S^¯/−^	S^¯^	–
*Solanum tuberoum* Amarilis	S^¯^	S^¯^	S^¯^	S^¯^	S^¯^	S^¯^	S^¯/−^	S^¯/−^	S^¯^	–

Host range: S: symptomless, + positive by ELISA, -/+: negative by ELISA, positive by PCR, +/+: positive by ELISA and PCR, N: not tested, LR: leaf rolling, MM: mild leaf mosaic, SM: severe leaf mosaic, CS: chlorotic spots. Control (+): SB-22 in *Physalis floridana* host, Control (-): healthy plant for each host. Samples (+/+), (-/+): reaction to test ELISA / RT-PCR.

An antiserum raised against FCiLV cross reacted with PYV-infected samples (from potato and yacon) as the antiserum raised against PYV [SB-22] cross reacted with FCiLV-infected samples. Neither PYV or FCiLV reacted with the AMV antiserum (Supplementary Fig. 1).

Potato plants corresponding to field samples collected between 2016–2018 (group 2) in which PYV was detected displayed varying symptomatology (supplementary Table 2) but many were co-infected with other viruses (mainly PVY, PVX, PVB). Plants infected only with PYV were symptomless or showed yellowing and were all confirmed to be positive by NCM ELISA of desiccated leaf tissue using PYV antibodies (not shown).

### 3.2 Sequence analysis and genome organization

Primers designed based on the sequence of contigs assembled from siRNAs were used to confirm the integrity of the assembled genomic RNAs. RNA1 of PYV [SB-22] consists of 3398 nucleotides (nt) and is monocistronic, encoding a protein containing Mtr and Hel domains sharing 87.6 % aa total sequence identity with FCiLV. RNA2 is 2427 nt long and encodes the RdRp protein, which shows 87.2 % aa identity with that of FCiLV and 25 % and 46.7 % with AMV and *Prune dwarf virus* (PDV; genus *Ilarvirus*, family *Bromoviridae*), respectively. No additional ORF was identified on RNA2 of PYV [SB-22] in contrast to cucumoviruses and ilarviruses of subgroup 1 and 2, where a 2b protein with RNA silencing suppression activity is found at this position (Li and Ding, 2001). RNA3 of PYV [SB-22] is 2381 nt long and is bicistronic encoding proteins with MP and CP domains. The putative MP is 35 kDa and shares a 70.7 %, 49.4 %, 20 % aa identity with MPs of FCiLV, PDV and AMV, respectively. The ORF CP has a predicted mass of 24 kDa. It shares 82.2 %, 47.6 % and 17 % aa sequence identity with FCiLV, PDV and AMV, respectively. The 3′-UTR is identical in the three genomic RNAs of PYV [SB-22] and as in other *Bromoviridae* it may fold into a stem-loop structure involved in CP binding and genome activation (Ansel‐McKinney et al., 1996).

For isolates from group 1, fragments were amplified and sequenced using primers PYV1F2/PYV123R designed to the Mtr and Hel motif in RNA1 (1626 nt), primers PYV2F2/PYV-123R located in the RdRp gene of RNA2 (1014 nt), and primer pairs PYV3F1/PYV3R1 (954 nt) and PYV3F2/PYV-123R (660 nt) corresponding to the MP and CP genes respectively on RNA3. These primers did not amplify the helicase gene in the yacon samples whereas the MP primers were not tested.

**Fig. 2:**
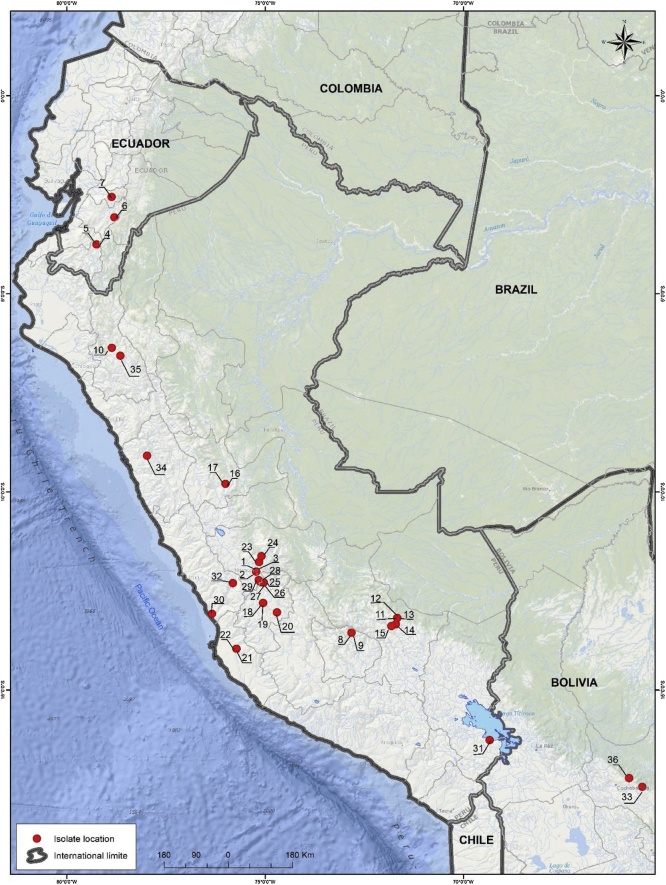
Map of sample collection sites in Peru, Ecuador, and Bolivia showing where PYV was detected (red spots). The numbers clustered around each collection site in this Figure indicate where each individual infected sample came and correspond to those in Table 1. The names marked on the map are those of the countries regional departments.

From the potato samples collected between 2016 and 2018 in Peru (group 2), 24 samples (2.4 %) were found to contain PYV like sequences (Table 1 and Fig. 2). For nine of those, the complete sequence of genomic RNA1, 2 and 3 were assembled, for 15 others only the sequence of genomic RNA3 could be fully assembled, whereas gaps remained in RNA1 and 2.

For the samples from the yacon germplasm collection (group 3), 3 out of 14 samples contained PYV like sequences and all three RNAs could be fully assembled, whereas all of them also contained contigs with >98 % identity to *Yacon necrotic mottle virus* (YNMoV) covering 94 % of the genome.

Analysis of the group 4 sample revealed 3 contigs with between 94–97 % nucleic acid identity to the RNA genome of PYV reported previously from Europe (Knierim et al., 2019). The sample was also found to contain Contigs with 98 % identity to YNMoV.

**Fig. 3:**
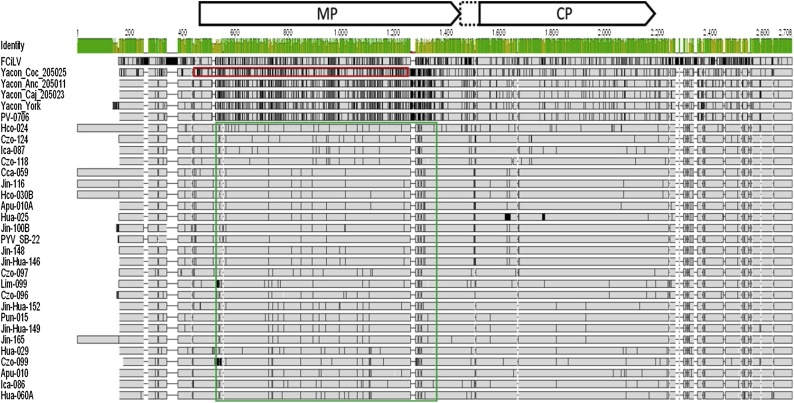
Schematic alignment of complete RNA3 sequences of PYV isolates and FCiLV. Grey boxes indicate aligned regions, black regions within them indicate dissimilarities from the consensus whereas lines indicate gaps in the alignment. Overall sequence identity levels are indicated in a bar graph for each position above the alignment. Nucleotide positions and the locations of MP and CP ORFs are indicated above the alignment, the dotted open box in CP ORF indicates 5′ extension found in 13 isolates. Red and green boxes indicate of recombinant fragments of unknown origin identified by RDP.

Alignment of all available RNA sequences revealed RNA3 (Fig. 3) showed relatively high variability (≥77 % nt identity) as compared to RNAs 1 and 2 (≥86 & 87 % nt identity respectively). ORF CP had a 5′ extension in 13 isolates as compared to isolate SB-22 and in addition, five potato isolates showed unusually long 5′ UTR sequence (412 nt) of RNA3 as compared to PYV [SB-22] and other isolates (258 nt) (Fig. 3). The sequence of the 5′ extension in these isolates showed no similarity to any other sequence and was only present in those 5 samples.

### 3.3 Phylogenetic and recombination analysis

**Fig. 4:**
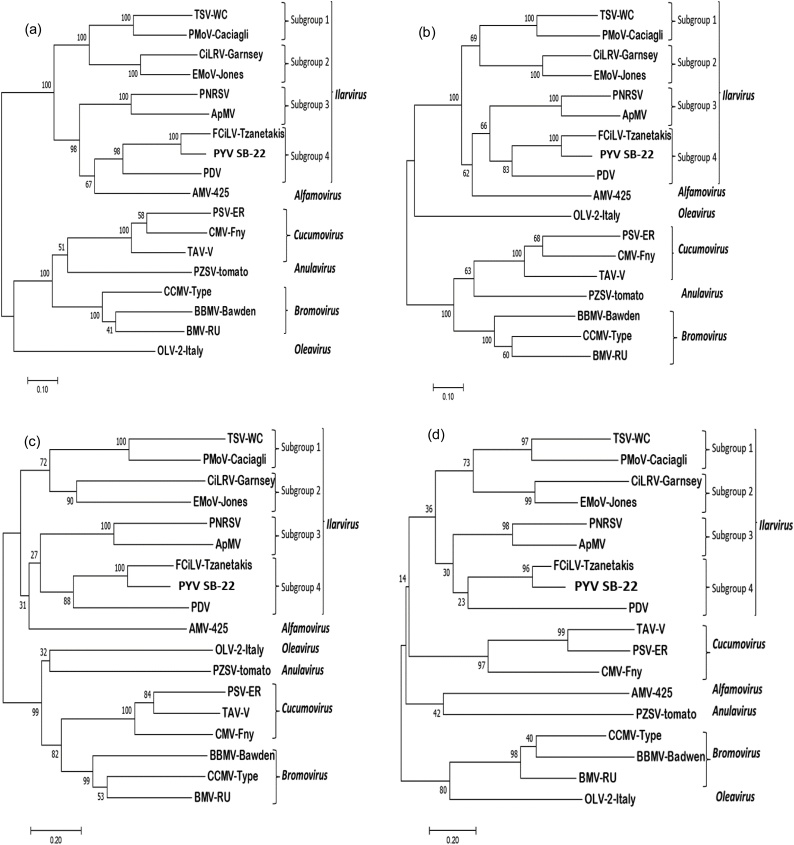
Phylogenetic tree of PYV [SB-22] with members of Family *Bromoviridae* using ORF nucleotides alignments. (a) Phylogenetic tree of ORF Mtr-Hel (b) Phylogenetic tree of ORF-RdRp and (c) Phylogenetic tree of ORF-CP and MP (d) Phylogenetic tree of ORF CP. Clustering method used was Neighbor Joining and molecular phylogenetic analysis by Maximum Likelihood method based on the Kimura 2-parameter model with 1000 bootstrap replications.

**Fig. 5:**
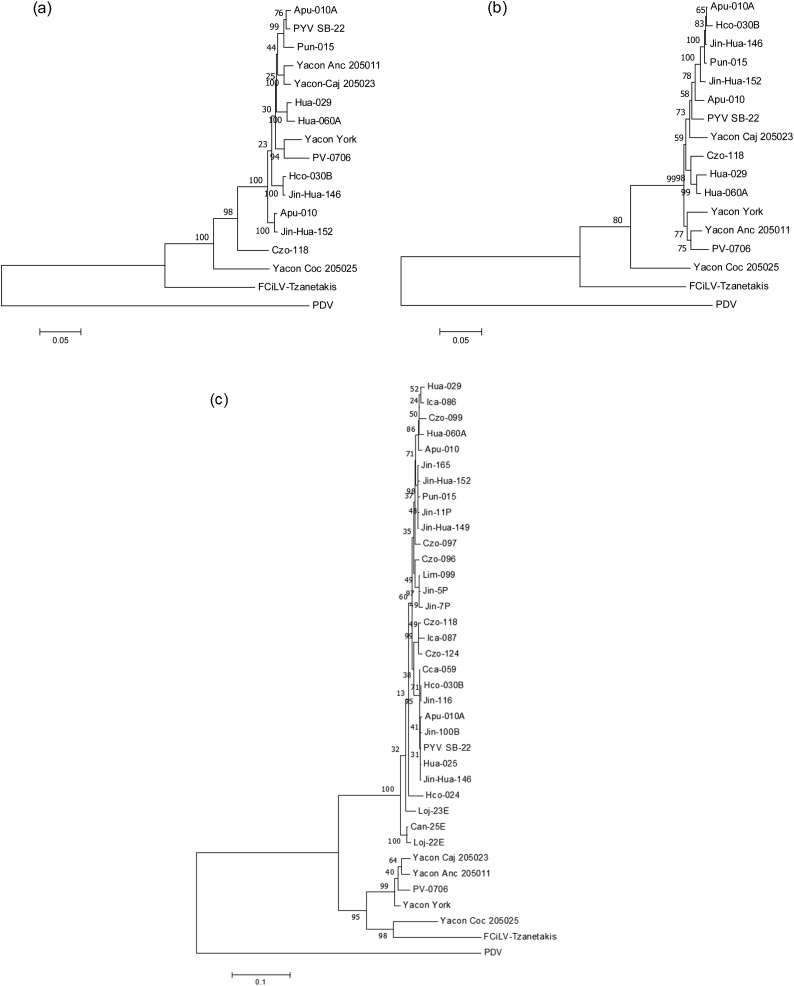
Phylogenetic tree of potato isolates using ORF nucleotides alignments. (a) Phylogenetic tree of ORF Mtr-Hel (b) Phylogenetic tree of ORF RdRp and (c) Phylogenetic tree of ORF CP and MP. Clustering method used was Neighbor Joining and molecular phylogenetic analysis by Maximum Likelihood method based on the Kimura 2-parameter model with 1000 bootstrap replications.

**Fig. 6:**
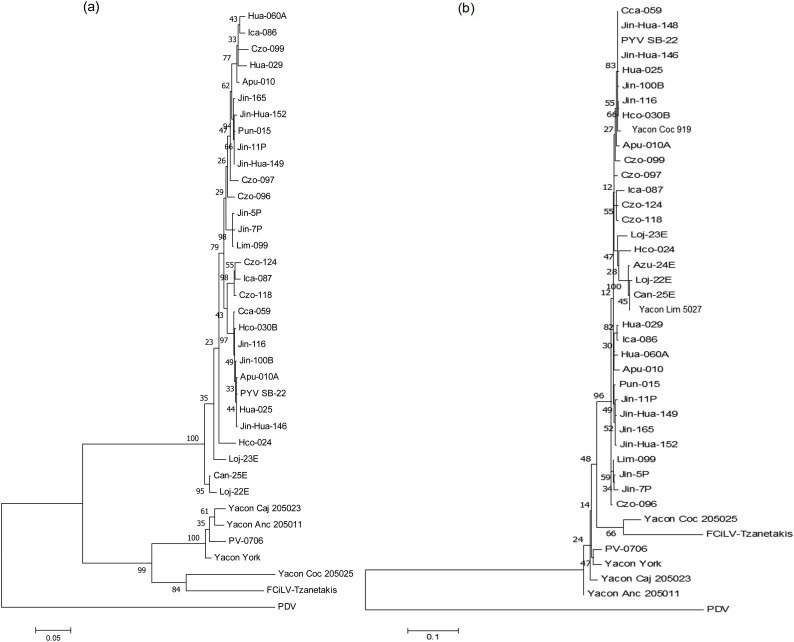
(a) Phylogenetic tree of ORF MP of potato isolates using nucleotides alignments and (b) Phylogenetic tree of ORF CP of potato and yacon isolates. Clustering method used was Neighbor Joining and molecular phylogenetic analysis by Maximum Likelihood method based on the Kimura 2-parameter model with 1000 bootstrap replications.

Phylogenetic analysis of the complete ORFs of all three RNA segments of PYV [SB-22] with representative members of the family *Bromoviridae* (Fig. 4) showed a well-supported cluster encompassing the genus *Ilarvirus* (King et al., 2012). AMV also falls into this cluster except when only the CP gene is used, in which case it is placed on a separate branch, close to unrelated other genera within the family. PYV [SB-22] fits in subgroup 4 when Mtr-Hel, RdRp, MP and CP domains amino acid sequences are used in the analysis supported by 100 % of bootstrap replicates. Analysis revealed a close relationship with FCiLV and PDV, the only members reported in this subgroup. Among the Ilarviruses, FCiLV was the closet relative of PYV [SB-22], with 82.2 % nucleotide and 87.2 % aa sequence identity with ORF RdRp. Moreover, the analysis confirmed the close relationship of AMV with some members of the genus *Ilarvirus* when analyzing the replication associated proteins. However, phylogenetic analysis of CP shows AMV in a separate cluster in the family *Bromoviridae* (Fig. 4d). Phylogenetic trees with PYV isolates from potato, yacon and pepino constructed with the complete ORF Mtr-Hel or ORF RdRp using nucleotides sequences formed an independent cluster different to FCiLV (Fig. 5a & b) to which they showed 80.6–81.6 % and 77.8–78.4 % nt identity respectively. On the other hand, the yacon isolate from Bolivia grouped separately from the other PYV samples with 87–89 % and 85.9–86.9 % nt identity to them for ORF Met-Hel and RdRp respectively, whereas all other isolates showed 95–96 %nt identity with each-other, with the exception of isolate Czo-118 which showed 91–92 % nt identity to the others for the Met-Hel ORF. Results from RNA3 on the other hand showed a more complex phylogeny with isolates from pepino and yacon together with FCiLV forming a separate clade from potato infecting isolates (Fig. 5c). The Bolivian isolate from yacon was distinct from the others, which formed separate a tight clade, grouping closer to FCiLV. However, when analyzing the ORFs for MP and CP separately (Fig. 6) trees with distinct topologies were generated, the one for MP being similar to that for the entire RNA3, whereas that for the CP grouped all isolates more closely together, even if the same grouping could still be recognized. Thus, for the MP, the yacon and pepino isolates showed only around 70 % nt identity to the potato isolates, whereas CP sequence all isolates showed >90 % nt identity to each-other except FCiLV which showed around 82 % nt identity to other isolates. The results suggest that the isolates from yacon and pepino might have MP proteins originating from a different origin than the potato isolates, perhaps as a result of recombination, since the 5´UTR showed similar levels of identity to other isolates as the CP (Fig. 3 and data not shown).

RDP4 analysis identified two recombination events. Considering FCiLV, Yacon Anc_205011, Yacon Caj_205023, Yacon York and PV-0706 isolates as non-recombinants, it detected a recombination of a fragment from an unknown source in isolate Yacon Anc_205011 between positions 449–1238, and in all potato isolates another fragment from unknown source between positions 504 and 1355 (Fig. 3). The recombination breakpoints were detected by 6 or seven methods implemented in RDP4 providing strong support for the recombination events (Supplementary Table 3).

## 4 Discussion

We report here the full genome characterization and detection of PYV isolates from potato and yacon from the Andean region and one yacon of unknown origin intercepted in the UK. Specific levels of sequence similarity for species demarcation among ilarviruses have not been defined (King et al., 2012), nevertheless, based on serological analyses, sequence similarity and host range we suggest that PYV and FCiLV are two distinct strains of the same Ilarvirus species. Antiserum against FCiLV or PYV [SB-22] are unable to distinguish between the two viruses and both can infect and produce similar symptoms in *C. quinoa* and *C. murale*. FCiLV and yacon isolates of PYV were unable to infect *Nicotiana* species which were infected by potato isolates. On the other hand, PYV from yacon were able to infect potato and it remains unknown whether PYV [SB-22] is able to infect *Fragaria chiloensis* or if FCiLV can infect potato. Because the name Potato yellowing virus was proposed for this virus first (Fuentes, 1992; Fuentes and Jayasinghe, 1993) it should be considered to rename FCiLV as a strain of PYV.

Phylogenetic analysis of the three RNAs of PYV indicate that the isolate Coc-205025 from yacon from Bolivia is the most distinct from the other isolates affiliating closer to FCiLV. At the same time, that particular isolate was identified as containing a recombinant fragment spanning the major part of the MP from an unknown origin as compared to the other isolates from yacon, pepino and FCiLV. Also, all the potato isolates were identified as containing a recombinant fragment of unknown origin, again spanning a large part of the MP. Thus, it appears that particularly the MP region has a propensity to recombine with other related viruses, which may provide the recombinants with novel biological properties. Indeed, if one considers RNA1, 2 and the non-recombinant regions of RNA3, then the potato, yacon (with the exception of Coc-205025) and pepino isolates cannot be clearly distinguished from each-other phylogenetically. It suggests that potato isolates might have obtained their ability to efficiently infect potato by acquiring an appropriate MP through recombination. Recombination and segment reassortment events are not uncommon in the evolutionary history of *Bromoviridae* including those between different genera within the family (Boulila, 2009; Cordoner et al., 2008). Thus the viruses in this family, including Ilarviruses and the viruses reported in this study, appear to utilize both strategies to evolve new biological properties such as host specificities.

Among the isolates biologically characterized from group 1, those from Ecuador cluster in a distinct phylogenetic clade when compared to Peruvian isolates using incomplete ORFs for Mtr-Hel and RdRP (Supplementary Fig. 2). A similar grouping could be observed through symptoms induced by isolates of these two groups in *P. floridana* (Fig. 1 and Table 3), although this was not consistent for all the isolates. In addition, the reaction using the PYV [SB-22] antiserum in the indicator plants that were mechanically inoculated from two asymptomatic accessions of yacon and the amplification of the sequences using ORF CP and ORF RdRp primers designed to PYV [SB-22] confirmed the presence of this virus in yacon samples. Although none of the primers designed to RNA1 of PYV [SB-22] were able to amplify corresponding sequences from the yacon samples of group 1, this could simply be a result of primer mismatches as those were readily identified with the sequences determined by sRSA from isolates in groups 2, 3 and 4.

The long 5′ untranslated sequence of 452 nt, identified in 5 potato isolates in this study, is not common in the members of the Ilarvirus group and their function is uncertain. To assure ourselves that these fragments were not mis-assemblies of sequences of plant origin or other viruses, we screened several other siRNA libraries of group 2 of the same and other cultivars and various combinations of virus infections for presence of similar sequences by alignment, but none other samples had siRNAs with similarity to the extended 5′ region. Previous reports have shown that the species *Triticum mosaic virus* (TriMV), family *Potyviridae*, had an unusually long 5′ untranslated region of 739 nt and was related to internal ribosome entry sites (IRES) elements for protein translation and the genome replication, both important for virulence (Tatineni et al., 2009).

PYV is the first member of the Ilarvirus group found to infect potato and yacon in the fields of the Andean region. Although the effect of PYV on potato or yacon production remains unknown, Jayasinghe and Chuquillanqui (1989) showed that PYV reduced the resistance to PLRV multiplication in potato, indicating indirect effects may also occur. Nevertheless, considering the limited infection frequency of less than 3 % found in our potato survey, we should consider it a virus of limited importance to potato production in Peru. A recently reported PYV isolate from pepino (*Solanum muricatum*) of unknown geographic origin in a German market (Knierim et al., 2019) indicates other crops can also be infected by this virus and thus should be considered in a broader context than just these two crops. The pepino isolate clearly groups with groups with the two yacon isolates from Peru and one from York, UK indicating they are related, in contrast to Coc-205025 from Bolivia which is clearly distinct. Nevertheless, we cannot say with any certainty that the German and UK isolates originated from Peru, as another isolate from Bolivia Coc-919 for which only partial sequences were determined, grouped with Peruvian isolates for the CP (Fig. 6b) and RdRp (not shown) sequences. However, with more extensive sequencing of isolates from different regions in the native range it might at one point be possible to trace routes of introduction of newly discovered viruses into a country.

Since its first report (Fuentes, 1992; Fuentes and Jayasinghe, 1993), PYV [SB-22] showed no serological relationship to AMV but other characteristics such as associated symptoms, morphology of their particles and aphid- and seed- transmission (Hemmati and McLean, 1977) indicated a certain degree of similarity between both viruses. AMV can infect potato and is regularly detected in fields in Peru (Valkonen et al., 1992b). At the genus level in the *Bromoviridae*, aphid-transmissibility is a property of AMV that sets it apart as the only member of the *Alfamovirus*. However, sequence analyses at genomic level show that AMV groups together with PYV, which is also aphid-transmitted, and other ilarviruses for all genomic ORFs excepting the CP (Fig. 2) supporting previous reports (Bol, 2005; Boulila, 2009; Codoner and Elena, 2008). A serological relationship between PYV and FCiLV but not AMV was confirmed in this study.

It has long been debated whether AMV (the sole of member of *Alfamovirus*) should be regarded as a member of the genus *Ilarvirus*. As noted previously, our phylogenetic analysis revealed a close relationship among ilarviruses and AMV for RNA1 and 2. The only property of AMV that sets it apart from the Ilarviruses is its aphid transmissibility, however PYV has also been shown to be aphid transmitted (Fuentes, 1992; Fuentes and Jayasinghe, 1993). On the other hand, even if RNA3 of AMV forms its own genetically distinct branch within *Bromoviridae*, it shares the requirement of CP for activation of replication with ilarviruses and this function can be substituted by CP from ilarviruses and vice versa (King et al., 2012) indicating close functional relationship among these viruses. Thus, there are exceedingly few arguments to maintain AMV in its own genus. Perhaps a new subgroup 4-R3A (RNA3 type AMV) can be considered to reclassify AMV to *Ilarvirus* as has been suggested by other authors previously (Boulila, 2009).

In conclusion our study has revealed the relative current frequency of PYV in potato in Peru and identified natural infections in yacon, expanding its known natural host range. It has also identified a relative plasticity in RNA3 with tentative extensions to the 5′ UTR, and 5′ of the CP gene in some isolates and two recombination events within the MP region being identified. This may be true for the genus Ilarvirus in general as the CP of AMV is also clearly a result of a recombination event, being the only protein distinguishing the current single member genus *Alfamovirus* apart from the genus *Ilarvirus* based on phylogenetic relationships.

## CRediT authorship contribution statement

**Rocio Silvestre:** Data curation, Formal analysis, Visualization, Writing - original draft, Writing - review & editing. **Segundo Fuentes:** Funding acquisition, Project administration, Data curation, Supervision, Writing - review & editing. **Roger Risco:** Investigation. **Alfredo Berrocal:** Investigation, Supervision. **Ian Adams:** Methodology, Formal analysis, Validation, Writing - review & editing. **Adrian Fox:** Funding acquisition, Methodology, Supervision, Writing - review & editing. **Wilmer J. Cuellar:** Funding acquisition, Methodology, Supervision, Writing - review & editing. **Jan Kreuze:** Conceptualization, Funding acquisition, Project administration, Methodology, Validation, Writing - review & editing.
