# Supplementary material for: Characterization of distinct strains of an aphid-transmitted ilarvirus (Fam. Bromoviridae) infecting different hosts from South America
Source: Virus Res. 2020 Jun;282:197944. doi: 10.1016/j.virusres.2020.197944 (PMC7221344; doi:10.1016/j.virusres.2020.197944)
Supplement: Supplementary file 1 [file mmc1.docx]

**Supplementary Table 1.** The RNA3 complete sequence of potato, yacon, pepino isolates of PYV and FCiLV used for recombination analysis.

| Sample Group | Isolate | Host | Genebank Number |
| --- | --- | --- | --- |
| Reference Isolate | PYV_SB-22 | *Solanum tuberosum* | MG672022 |
| Group 2 | Apu-010 | *Solanum tuberosum* | MN527471 |
|  | Apu-010A | *Solanum tuberosum* | MN527474 |
|  | Cca-059 | *Solanum tuberosum* | MN527517 |
|  | Czo-096 | *Solanum tuberosum* | MN527509 |
|  | Czo-097 | *Solanum tuberosum* | MN527508 |
|  | Czo-099 | *Solanum tuberosum* | MN527506 |
|  | Czo-124 | *Solanum tuberosum* | MN527512 |
|  | Czo-118 | *Solanum tuberosum* | MN527477 |
|  | Hco-024 | *Solanum tuberosum* | MN527519 |
|  | Hco-030B | *Solanum tuberosum* | MN527495 |
|  | Hua-025 | *Solanum tuberosum* | MN527514 |
|  | Hua-029 | *Solanum tuberosum* | MN527480 |
|  | Hua-060A | *Solanum tuberosum* | MN527483 |
|  | Ica-086 | *Solanum tuberosum* | MN527505 |
|  | Ica-087 | *Solanum tuberosum* | MN527511 |
|  | Jin-100B | *Solanum tuberosum* | MN527513 |
|  | Jin-116 | *Solanum tuberosum* | MN527518 |
|  | Jin-165 | *Solanum tuberosum* | MN527516 |
|  | Jin-Hua-146 | *Solanum tuberosum* | MN527486 |
|  | Jin-Hua-148 | *Solanum tuberosum* | MN527515 |
|  | Jin-Hua-149 | *Solanum tuberosum* | MN527507 |
|  | Jin-Hua-152 | *Solanum tuberosum* | MN527489 |
|  | Lim-099 | *Solanum tuberosum* | MN527510 |
|  | Pun-015 | *Solanum tuberosum* | MN527492 |
| Group 3 | Yacon_Anc-205011 | *Smallanthus sonchifolius* | MN527504 |
|  | Yacon_Caj-205023 | *Smallanthus sonchifolius* | MN527498 |
|  | Yacon_Coc-205025 | *Smallanthus sonchifolius* | MN527501 |
| Group 4 | Yacon_York | *Smallanthus sonchifolius* | MN548139 |
| - | PV-0706 | *Solanum muricatum* | MH937420 |
| - | FCiLV | *Fragaria chiloensis* | AY707772 |
