# Supplementary material for: Characterization of distinct strains of an aphid-transmitted ilarvirus (Fam. Bromoviridae) infecting different hosts from South America
Source: Virus Res. 2020 Jun;282:197944. doi: 10.1016/j.virusres.2020.197944 (PMC7221344; doi:10.1016/j.virusres.2020.197944)
Supplement: Supplementary file 2 [file mmc2.docx]

**Supplementary Table 2.** Symptoms observed in the field and viruses detected by small RNA sequencing and assembly of potato isolates collected between 2016 and 2018 in Peru (group 2 samples).

| Sample code | Department | Viruses detected | Symptoms | Cultivar |
| --- | --- | --- | --- | --- |
| Apu-10 | Apurimac | PYV, PVY | Curling?, yellowing | - |
| Apu-010A | Apurimac | PYV, PVB, PVX, PVY | Mosaic | - |
| Cca-059 | Cajamarca | PYV, PLRV, PVX | Yellow, mosaic | Yungay |
| Czo-096 | Cuzco | PYV, PVB, PVY, PVV | Leaf roll, purpling | Canchan |
| Czo-097 | Cuzco | PYV, PVB, PVY, PVV | Chlorosis | Canchan |
| Czo-099 | Cuzco | PYV, PVV, PVY | Curling, leaf reduction, stunting, chlorosis | Canchan |
| Czo-118 | Cuzco | PYV, PVX, PVY, Torrado | Curling, stunting, mottle | Cica |
| Czo-124 | Cuzco | PYV, PVY | No symptoms / yellowing bottom part?? | Yungay |
| Hco-024 | Huanuco | PYV, PVX, PVY | Rugosity, mosaic, stunting? | Canchan/Blanca |
| Hco-030B | Huanuco | PYV, PVX, PVY, APMoV, Comovirus | Rugosity, mosaic, stunting | Canchan/Blanca |
| Hua-025 | Huancavelica | PYV, PVB, PVX | Mosaic, rugosity, curling | Ccompis/Huayro |
| Hua-029 | Huancavelica | PYV, PVB | Mosaic, rugosity, curling | Ccompis/Huayro |
| Hua-060A | Huancavelica | PYV, PVB, PVX | Yellowing | - |
| Ica-086 | Ica | PYV, FCiLV | No symptoms | Canchan |
| Ica-087 | Ica | PYV, FCiLV | Chlorosis?, leaf reduction? | Canchan |
| Jin-100B | Junin | PYV, PVY | Chlorosis?, stunting? | Andina |
| Jin-116 | Junin | PYV, PVX, PVY | Chlorosis, mottle | Yungay |
| Jin-165 | Junin | PYV, PVX | Mottle? | Yungay |
| Jin-Hua-146 | Junin - Huancavelica | PYV | Curling, yellowing? | Andina |
| Jin-Hua-148 | Junin - Huancavelica | PYV | Curling, yellowing | Andina |
| Jin-Hua-149 | Junin - Huancavelica | PYV | Curling?, yellowing | Andina |
| Jin-Hua-152 | Junin - Huancavelica | PYV, PVX | Mottle? | Yungay |
| Lim-099 | Lima | PYV, FCiLV, APMoV, Comovirus, Nepovirus | Mosaic, rugosity | Unica |
| Pun-015 | Puno | PYV, FCiLV, PVB, PVX, PVV, PVA | Yellowing, calico | Ccompis |
